# Supplementary material for: A network dynamical simulation model for the study of antibiotic resistance in microbial communities
Source: Gut Microbes. 2026 Sep 19;18(1):2734703. doi: 10.1080/19490976.2026.2734703 (PMC13596919; doi:10.1080/19490976.2026.2734703)
Supplement: Supplementary file 1 [file KGMI_A_2734703_SM9053.zip › supplementary_file_1/Figures/ws_pct_delta_n_edges.pdf]

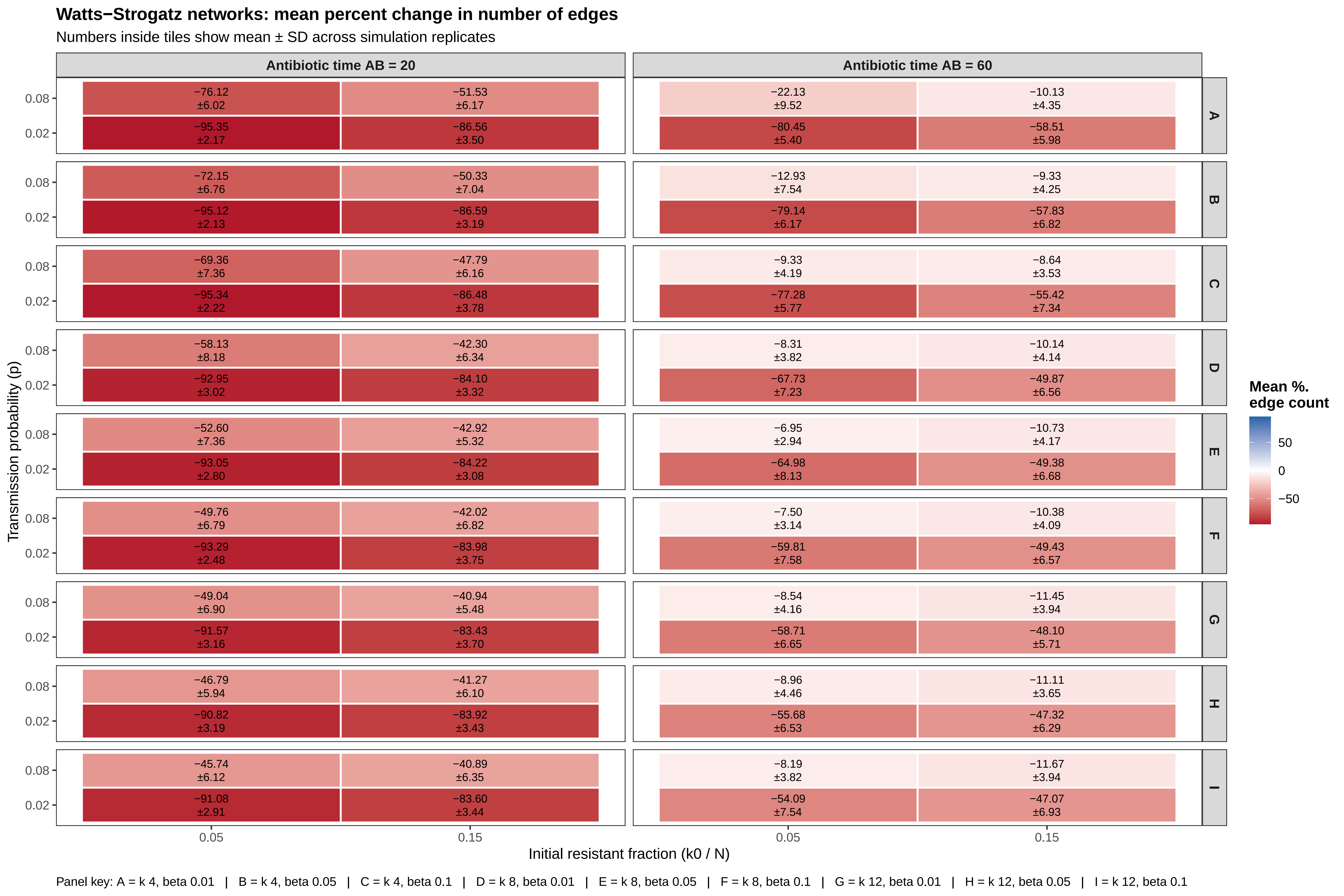
Watts−Strogatz networks: mean percent change in number of edges
Numbers inside tiles show mean ± SD across simulation replicates
Antibiotic time AB = 20
Antibiotic time AB = 60
A
−76.12 ±6.02
−51.53 ±6.17
−95.35 ±2.17
−86.56 ±3.50
−22.13 ±9.52
−10.13 ±4.35
−80.45 ±5.40
−58.51 ±5.98
B
−72.15 ±6.76
−50.33 ±7.04
−95.12 ±2.13
−86.59 ±3.19
−12.93 ±7.54
−9.33 ±4.25
−79.14 ±6.17
−57.83 ±6.82
C
−69.36 ±7.36
−47.79 ±6.16
−95.34 ±2.22
−86.48 ±3.78
−9.33 ±4.19
−8.64 ±3.53
−77.28 ±5.77
−55.42 ±7.34
D
−58.13 ±8.18
−42.30 ±6.34
−92.95 ±3.02
−84.10 ±3.32
−8.31 ±3.82
−10.14 ±4.14
−67.73 ±7.23
−49.87 ±6.56
E
−52.60 ±7.36
−42.92 ±5.32
−93.05 ±2.80
−84.22 ±3.08
−6.95 ±2.94
−10.73 ±4.17
−64.98 ±8.13
−49.38 ±6.68
F
−49.76 ±6.79
−42.02 ±6.82
−93.29 ±2.48
−83.98 ±3.75
−7.50 ±3.14
−10.38 ±4.09
−59.81 ±7.58
−49.43 ±6.57
G
−49.04 ±6.90
−40.94 ±5.48
−91.57 ±3.16
−83.43 ±3.70
−8.54 ±4.16
−11.45 ±3.94
−58.71 ±6.65
−48.10 ±5.71
H
−46.79 ±5.94
−41.27 ±6.10
−90.82 ±3.19
−83.92 ±3.43
−8.96 ±4.46
−11.11 ±3.65
−55.68 ±6.53
−47.32 ±6.29
I
−45.74 ±6.12
−40.89 ±6.35
−91.08 ±2.91
−83.60 ±3.44
−8.19 ±3.82
−11.67 ±3.94
−54.09 ±7.54
−47.07 ±6.93
0.08
0.02
Transmission probability (p)
0.05
0.15
Initial resistant fraction (k0 / N)
Mean %. edge count
50
0
−50
Panel key: A = k 4, beta 0.01 | B = k 4, beta 0.05 | C = k 4, beta 0.1 | D = k 8, beta 0.01 | E = k 8, beta 0.05 | F = k 8, beta 0.1 | G = k 12, beta 0.01 | H = k 12, beta 0.05 | I = k 12, beta 0.1
